# HMGB1-mediated autophagy regulates sodium/iodide symporter protein degradation in thyroid cancer cells

**DOI:** 10.1186/s13046-019-1328-3

**Published:** 2019-07-22

**Authors:** Wenwen Chai, Fanghua Ye, Li Zeng, Yanling Li, Liangchun Yang

**Affiliations:** 1 Department of Nuclear Medicine, Hunan Cancer Hospital, Changsha, Hunan 410008 People’s Republic of China; 2 Department of Pediatrics, Xiangya Hospital, Central South University, Changsha, Hunan 410008 People’s Republic of China

**Keywords:** HMGB1, Autophagy, NIS, AMPK, mTOR

## Abstract

**Background:**

Sodium/iodide symporter (NIS)-mediated iodide uptake plays an important physiological role in regulating thyroid gland function, as well as in diagnosing and treating Graves’ disease and thyroid cancer. High-mobility group box 1 (HMGB1), a highly conserved nuclear protein, is a positive regulator of autophagy conferring resistance to chemotherapy, radiotherapy and immunotherapy in cancer cells. Here the authors intended to identify the role of HMGB1 in Hank’s balanced salt solution (HBSS)-induced autophagy, explore NIS protein degradation through a autophagy-lysosome pathway in thyroid cancer cells and elucidate the possible molecular mechanisms.

**Methods:**

Immunohistochemical staining and reverse transcription-polymerase chain reaction (RT-PCR) were performed for detecting the expression of HMGB1 in different tissues. HMGB1 was knocked down by lentiviral transfection in FTC-133/TPC-1 cells. Autophagic markers LC3-II, p62, Beclin1 and autophagosomal formation were employed for evaluating HMGB1-mediated autophagy in HBSS-treated cells by Western blot, immunofluorescence and electron microscopy. Western blot, quantitative RT-PCR and gamma counter analysis were performed for detecting NIS expression and iodide uptake in HMGB1-knockdown cells after different treatments. The reactive oxygen species (ROS) level, ROS-mediated LC3-II expression and HMGB1 cytosolic translocation were detected by fluorospectrophotometer, flow cytometry, Western blot and immunofluorescence. HMGB1-mediated AMPK, mTOR and p70S6K phosphorylation (p-AMPK, p-mTOR & p-p70S6K) were detected by Western blot. Furthermore, a nude murine model with transplanted tumor was employed for examining the effect of HMGB1-mediated autophagy on imaging and biodistribution of ^99m^TcO4^−^. NIS, Beclin1, p-AMPK and p-mTOR were detected by immunohistochemical staining and Western blot in transplanted tumor samples.

**Results:**

HMGB1 was a critical regulator of autophagy-mediated NIS degradation in HBSS-treated FTC-133/TPC-1 cells. And HMGB1 up-regulation was rather prevalent in thyroid cancer tissues and closely correlated with worse overall lymph node metastasis and clinical stage. HMGB1-knockdown dramatically suppressed autophagy, NIS degradation and boosted iodide uptake in HBSS-treated cells. Moreover, HBSS enhanced ROS-sustained autophagy and promoted the cytosolic translocation of HMGB1. A knockdown of HMGB1 suppressed LC3-II conversion and NIS degradation via an AMPK/mTOR-dependent signal pathway through a regulation of ROS generation, rather than ATP. Furthermore, these data were further supported by our in vivo experiment of xenografts formed by HMGB1 knockdown cells reverting the uptake of ^99m^TcO4^−^ as compared with control shRNA-transfected cells in hunger group.

**Conclusions:**

Acting as a critical regulator of autophagy-mediated NIS degradation via ROS/AMPK/mTOR pathway, HMGB1is a potential intervention target of radioiodine therapy in thyroid cancer.

**Electronic supplementary material:**

The online version of this article (10.1186/s13046-019-1328-3) contains supplementary material, which is available to authorized users.

## Background

Active iodide accumulation marks an initial step in the biosynthesis of iodine-containing thyroid hormones in thyroid follicular cell [1]. As an integral glycoprotein located at basolateral plasma membrane, sodium/iodide symporter (NIS) might efficiently mediate active iodide accumulation into thyroid follicular cell [2]. Application of radioactive iodide isotopes has brought great advances in the diagnosis and treatment of thyroid cancer and provide a molecular basis for justifying radioiodide treatment for extrathyroidal malignancies [3, 4]. Radioiodide treatment for ablating thyroid cancer metastases and remnants after thyroidectomy has been the most successful targeted internal radiotherapy [5]. Its effectiveness is ultimately dependent on functional NIS expression at plasma membrane of tumor cells since deficient radioiodide accumulation is a major cause of treatment failure [6]. NIS gene expression was frequently down-regulated in thyroid cancer and became almost totally silenced in poorly differentiated and anaplastic thyroid carcinomas [7, 8]. Most studies demonstrated that iodide uptake by thyrocytes was basically dependent upon NIS transcriptional regulation [8, 9]. NIS post-translational regulation occurred through its cellular traffic and stability and autophagy-lysosome pathway-mediated degradation, thus consequently affecting iodide uptake by thyrocytes [10, 11]. Although several transcriptional and posttranscriptional mechanisms have been postulated for explaining a repression of NIS gene expression in thyroid tumors, further details regarding NIS-mediated resistance to radioiodide therapy remain unknown.

As a natural process in which subcellular membranes undergo dynamic morphological changes leading to the degradation of cellular proteins and cytoplasmic organelles, autophagy has been implicated in many physiological and pathophysiological conditions [12]. It became typically up-regulated when cellular survival required intracellular nutrients and energy [13]. Autophagy is important in the regulation of cancer development and progression and in determining the response of tumor cells to anticancer therapy. It might facilitate removal of damaged proteins or organelles during drug treatment in cancer cells and provide spare parts from degraded cellular components [12]. Autophagy has also been implicated in the development and treatment of thyroid cancer [14–16]. Heightened autophagy after multiple antitumor treatment regimens in thyroid cancer could enable tumor cells to be more sensitive to anticancer therapy [15]. In contrast, pharmacologic or genetic inhibition of autophagy enhanced antitumor activity of vemurafenib, a selective and well-tolerated BRAF inhibitor, in thyroid cancer [16]*.* Protein degradation could occur through ubiquitin-proteasome system (UPS) or autophagy-lysosome pathway [17]. Recent studies have confirmed that NIS protein was degraded in thyroid or mammary cells after an activation of autophagy-lysosome pathway [11, 18]. However, the underlying molecular mechanism of autophagy regulating NIS degradation in thyroid cancer cells has remained illusive.

As a dynamic nuclear protein with vital roles during gene transcription, chromatin remodeling, DNA recombination and repair [19], HMGB1 is also translocated into cytosol and extracellular space by multiple cellular stressors (e.g. protein aggregates, radiation, oxidation, chemotherapy and intracellular pathogens) [20–22]. Dysfunction of intracellular and extracellular HMGB1 has been implicated in the pathogenesis of infections, cancer, neurodegeneration, aging and cardiac disease [23–25]. An overexpression of HMGB1 was observed in leukemia, osteosarcoma, breast cancer, lung cancer and prostate cancer and there was also a strong association with their progression or prognosis [20, 24, 25]. During tumor development and treatment, HMGB1 might play paradoxical roles in promoting both cell survival and death by regulating multiple signaling pathways, including immunity, genomic stability, proliferation, metastasis, metabolism, apoptosis and autophagy [20, 26, 27]. Our previous studies have shown that HMGB1 plays an important role in leukemic pathogenesis and chemotherapeutic resistance [24, 27, 28]. As a positive regulator of autophagy, intracellular HMGB1 interacts with Beclin-1 in leukemic cells leading to autophagosomal formation, an alternative mechanism of resistance to leukemic therapies [27]. Although these studies have enriched our understanding of the role of HMGB1 in regulating autophagy and autophagy-related chemoresistance in leukemic cells, its possible role in the regulation of NIS degradation and radioiodide therapy by autophagy in thyroid cancer cells is unknown.

In the present study, we examined the expression of HMGB1 in various thyroid cancer cell lines and patient samples, HMGB1 involvement in autophagy, its influence on NIS degradation and iodide uptake of thyroid cancer cells and its relationship with ROS/AMPK/mTOR pathway. The objective was to obtain more insights into the function of HMGB1-mediated autophagy regulating NIS protein degradation.

## Materials and methods

### Reagents and cell culture

3-methyladenine (3-MA; #M9281), N-acetylcysteine (NAC; #A0737), diphenyleneiodonium chloride (DPI; #D2926) and rapamycin (#R8781) were supplied by Sigma-Aldrich (St. Louis, MO, USA); spautin-1 (#C3430) from Cellagen Technology (San Diego, CA, USA); TPC-1, K1, KTC-1 and FRO from American Type Culture Collection (Manassas, VA, USA); human bronchial epithelial cell HBE, normal human thyroid cell HT-ori3, acute myeloid leukemia cell HL-60 and chronic myelogenous leukemia cell K562 from Xiangya School of Medicine Type Culture Collection (Changsha, China). Following the procedures outlined previously [4], we utilized FTC-133 and TPC-1 thyroid cancer cell lines (Additional file 1: Figure S1) stably expressing human NIS (hNIS). HL-60 and K562 cells were cultured in RPMI-1640 medium (Invitrogen, San Diego, CA) and FTC-133, TPC-1, K1, KTC-1, FRO, HBE and HT-ori3 cells in Dulbecco’s modified Eagle’s medium (DMEM; Hyclone, Thermo Fisher Scientific, Waltham, MA, USA) supplemented with 10% heat-inactivated fetal bovine serum (FBS; Thermo Fisher Scientific Inc., Waltham, MA) and 1% antibiotics (100 U/mL penicillin G & 100 mg/mL streptomycin) at 37 °C in a cell incubator with 5% CO_2_ (Thermo Fisher Scientific Inc., USA).

### Patients and samples collection

The study protocol was approved by the Ethics Committee of Hunan Cancer Hospital. And written informed consents were obtained from all participants before using clinical samples for researches. Thyroid cancer (*n* = 36), thyroid adenoma (*n* = 20), simple goiter (*n* = 17) and normal thyroid tissues (*n* = 15) were collected from January 2015 to December 2018. The diagnosis, stage and risk status of thyroid cancer were assessed in accordance with the criteria of National Comprehensive Cancer Network. The general clinical and laboratory profiles of patients were summarized in Table 1. Tissue samples were immediately immersed into liquid nitrogen after surgical removal and preserved at − 80 °C. Frozen tumor tissues and matching normal tissues from 88 cases were subjected to mRNA extraction for reverse transcription-polymerase chain reaction (RT-PCR). Samples from 36 patients with complete clinicopathological and follow-up information were selected for assessing the correlation of HMGB1 expression with clinical features based on RT-PCR analysis.

**Table 1:** Relationship between HMGB1 expression and clinicopathologic features in a validated cohort

Clinicopathologic features	Low expression, *n* = 18 (%)	High expression, n = 18 (%)	*P*-value
Age (years)			
< 45	9 (50%)	10 (55.6%)	*P* = 0.738
≥ 45	9 (50%)	8 (44.4%)	
Gender			
Male	7 (66.7%)	8 (22.2%)	*P* = 0.597
Female	11 (33.3%)	10 (77.8%)	
Tumor size (cm)			
< 1	12 (66.7%)	6 (33.3%)	*P* = 0.046
≥ 1	6 (33.3%)	12 (66.7%)	
Lymph node metastasis			
No	13 (72.2%)	6 (33.3%)	*P* = 0.019
Yes	5 (27.8%)	12 (66.7%)	
Histological type			
PTC	9 (50%)	10 (55.6%)	P = 0.738
FTC	9 (50%)	8 (44.4%)	
Clinical stage			
I + II	13 (72.2%)	7 (38.9%)	*P* = 0.044
III + IV	5 (27.8%)	11 (61.1%)	

### Lentivirus, plasmid transfection and RNA interference

HMGB1 small hairpin RNA (shRNA) lentiviral knockdown (GeneCopoeia, Guangzhou, China) or shRNA non-target control (NTC) were packaged with HIV-based packaging mix (GeneCopoeia) for infecting FTC-133/TPC-1 cells for establishing cells constitutively repressing HMGB1. Stable clones were selected by puromycin. The HMGB1 shRNA oligonucleotide sequences were as follows: HMGB1 shRNA: 5′ - CCGGGCAGATGACAAGCAGCCTTATCTCGAGATAAGGCTGCTTGTCATCTGCTTTTT-3′; Non-silencing shRNA (control shRNA) were used as mock-transfected controls (target sequence: TTCTCCGAACGTGTCACGT). Human SOD1-siRNA (target sequence: GGUGGAAAUGAAGAAAGUAC) was transfected with Lipofectamine RNAiMAX reagent (for siRNA) according to the manufacturer’s instructions (Invitrogen). After incubating 48-h with RNA interference (RNAi), culture medium was replaced before subsequent treatments.

### Reverse transcription polymerase chain reaction (RT-PCR)

Following the procedures outlined previously [24], total RNA was isolated from patient tissues and FTC-133 cells using Trizol reagent (Invitrogen, USA) according to the manufacturer’s instructions. RNA concentration and purity were measured with a spectrophotometer at A260 and A260/280 respectively. And RNA was reverse-transcribed into cDNA using a Primescript™ RT reagent kit (Invitrogen) according to the manufacturer’s instructions. The sequences of primers used were as follows: for β-actin: forward, 5′-TCCTTCCTGGGCATGGAGTC-3′ and reverse 5′-GTAACGCAACTAAGTCATAGTC-3′; for HMGB1: forward, 5′-TTTCAAACAAAGATGCCACA-3′ and reverse, 5′-GTTCCCTAAACTCCTAAGCAGATA-3′; for hNIS: forward, 5′-GTCGTGGTGATGCTAAGTGGC-3′ and reverse, 5′-ATTGATGCTGGTGGATGCTGT-3′. β-Actin was used as an internal control for evaluating the relative expressions of HMGB1 and hNIS. The conditions for polymerase chain reaction (PCR) to HMGB1 were as follows: denaturation at 94 °C for 2 min, followed by 30 cycles of 94 °C for 30 s, 56 °C for 30 s (β -actin: 50 °C for 30 s), 72 °C for 30 s and then by a 5 min elongation at 72 °C. The conditions for polymerase chain reaction (PCR) to hNIS were: denaturation at 94 °C for 2 min, followed by 30 cycles of 94 °C for 30 s, 61 °C for 30 s (β-actin: 50 °C for 30 s), 72 °C for 30 s and then by a 2 min elongation at 72 °C. PCR products were analyzed with 1.0% agarose gel electrophoresis, ethidium bromide (EB) staining, photographed and scanned using Band Leader software for gray-scale semiquantitative analysis.

### Quantitative real-time PCR (qRT-PCR)

TRIzol (Invitrogen) was employed for isolating total cell RNA and cDNA synthesized with PrimeScript™ RT Master Mix (Takara Biomedical Technology Co., Ltd., Beijing, China). A 7900 Real-Time PCR System (Applied Biosystems, Foster City, CA) with AceQ qPCR SYBR Green Master Mix (High ROX Premixed Vazyme Biotech Co., Ltd., Nanjing, China) was used for performing quantitative PCR (qPCR). Relative mRNA expression was standardized using the housekeeping gene β-actin forward (5′-CATTAAGGAGAAGCTGTGCT-3′) and reverse (5′-GTTGAAGGTAGTTTCGTGGA-3′). The following human primers were used in this study: NIS forward (5′-GCGTGGCTCTCTCAGTCAA-3′) and reverse (5′-GCGTCCATTCCTGAGCTG-3′). Cycling conditions were as follows: 98 °C for 2 min, followed by 40 cycles of 98 °C for 10 s, 60 °C for 10 s and 68 °C for 30 s. The relative gene expressions were calculated by comparative CT method. Samples were examined in triplicate.

### Antibodies, preparation of subcellular fractions and Western blot

The following commercially available antibodies were used: murine anti-HMGB1 (#H00003146-M08), rabbit anti-Beclin-1 (#NB500–249) and rabbit anti-LC3 (#NB600–1384) were obtained from Novus (Littleton, CO, USA); rabbit antibodies against superoxide dismutase 1 (SOD1)(# ab51254), tubulin (#ab18251), fibrillarin (#ab4566) and actin (#ab3280) from Abcam (Cambridge, MA, USA); rabbit antibodies against phospho(p)-mTORS2448 (#2971), mTOR (#2972), p-p70S6KT389(#9205), p70S6K (#9202), p-AMPKαT172 (#9282), AMPKα (#2532), horseradish peroxidase (HRP)-conjugated goat anti-rabbit IgG (7074) and HRP-conjugated goat antimouse IgG (7072) from Cell Signaling Technology (Danvers, MA, USA); antibody to p62 (#Sc-28359) from Santa Cruz Biotechnology (Dallas, TX, USA); antibody against human NIS (#ab83816) from Abcam (Cambridge, MA, USA). NIS has a hyper-glycosylated form of molecular weight of approximately 70 to 90 kDa and a hypo-glycosylated form of 60 kDa. The hyper-glycosylated NIS represents the mature and main form of iodide-pumping NIS on plasma membrane [29]. A hyper-glycosylated form of molecular weight of 69 kDa was detected in the present study.

Cells were rinsed with PBS, collected and resuspended in lytic buffer (Beyotime, Beijing, China) and maintained on ice for 15 min. Cytosolic/nuclear extracts and total cellular lysates were prepared using the NE-PER nuclear and cytoplasmic extraction kit (Piece, Rockford, USA) according to the manufacturer’s instructions. Protein concentrations of the extracts were measured with BCA assay (Pierce, Rockford, USA) and equalized with the extraction reagents. Whole cell lysates were separated by 8, 10% or 12% sodium dodecyl sulfate-polyacrylamide gel electrophoresis (SDS-PAGE) and subsequently electrophoretically transferred onto polyvinylidene difluoride (PVDF) blotting membranes (Beyotime, Beijing, China). The membranes were blocked with 5% non-fat dry milk in Tris-buffered saline containing Tween 20 (TBST; 50 mM Tris [pH 7.5], 100 mM NaCl, 0.15% Tween-20), incubated with diluted primary antibodies for 12 h at 4 °C, and rinsed thrice with TBST for 10 min. Then the membranes were incubated with different secondary antibodies for 12 h at 4 °C and hybridization was detected by enhanced chemiluminescent reagents (Pierce, Waltham, MA) after rinsing thrice with TBST for 10 min. Membranes were exposed to radiographic film and the expression levels of targeted proteins quantified. A BandScan 5.0 system was employed for quantifying and analyzing specific bands via Western blot.

### Immunofluorescent analysis

Cells were fixed in 4% formaldehyde for 30 min at room temperature prior to cell permeabilization with 0.1% Triton X-100 (4 °C, 10 min). After saturating with PBS containing 2% bovine serum albumin for 1 h at room temperature, the samples were processed for immunofluorescence with anti-LC3B antibody (L7543, Sigma) followed by Alexa Fluor 488-conjugated immunoglobulin and DAPI (Sigma). Between all incubation steps, cells were rinsed thrice for 3 min with PBS containing 0.2% bovine serum albumin. Fluorescent signals were analyzed using an Olympus Fluoview 1000 confocal microscope (Olympus Corp, Tokyo, Japan).

### Immunohistochemistry

Both patient samples and murine tumors were fixed in 10% formalin for 24 h. After dehydration and paraffin embedding, the specimens were sliced into 5-μm thick sections by a microtome (Leica, Wetzlar, Germany) and mounted on glass slides. The expressions of HMGB1, Beclin1 and NIS were measured by immunostaining. After deparaffinization and rehydration, the sections were pressure-cooked for 2 min in an antigen retrieval buffer (0.01 M citrate buffer, pH 6.0) for unmasking antigens. Then the sections were incubated with murine anti-rat HMGB1, Beclin1 and NIS monoclonal antibodies (1:200, Cat. No. 113802, Biolegend, San Diego, CA) at 4 °C overnight respectively, followed by biotinylated anti-murine IgG secondary antibody (ZSGB-bio, Beijing, China) for 1 h at 37 °C and streptavidin-HRP (ZSGB-bio) for 30 min at 37 °C. Furthermore, HRP substrate DAB (3, 3-diaminobenzidine; ZSGB-bio, China) was utilizing for developing and visualizing the immunostained samples whereas cell nuclei were counter-stained with hematoxylin. Images were acquired with an Olympus BX51 microscope for assessing the proportion of positively stained cells.

### Electron microscopy

Cells were collected and fixed in 2.5% glutaraldehyde for at least 3 h. Then the cells were treated with 2% paraformaldehyde at room temperature for 60 min and 0.1% glutaraldehyde in 0.1 M sodium cacodylate for 2 h and followed by post-fixing with 1% OsO4 for 1.5 h. After a second rinsing, cells were dehydrated with graded acetone and finally embedded in Quetol 812. Ultrathin sections were observed under an H7500 electron microscope (Hitachi, Tokyo, Japan).

### Determination of ROS generation

The intracellular alterations of ROS were determined by measuring the oxidative conversion of cell-permeable 2′,7′-dichlorofluorescein diacetate (DCFH-DA) into fluorescent dichlorofluorescein (DCF) on a fluorospectrophotometer (F4500, Hitachi, Tokyo, Japan) according to the methods described previously [22] or by flow cytometry. In brief, cells exposed to different treatments were collected, rinsed with D-Hank’s buffer and incubated with DCFH-DA at 37 °C for 30 min. Then DCF fluorescence of 20,000 cells was detected by a fluorospectrophotometer at an excitation wavelength of 488 nm and an emission wavelength of 535 nm. Or fluorescent intensity of DCF was measured by flow cytometry. Incremental production of ROS was expressed as a percentage of control.

### Measurement of intracellular ATP level

The cellular levels of ATP were detected by Enhanced ATP Assay Kit (Beyotime Biotechnology) according to the manufacturer’s instructions. Briefly assay buffer was gently mixed with substrate at room temperature. Then mixed reagent 100 μl was loaded into each well and incubated for 15 min at room temperature. Then luminescence was measured by a microplate reader (Beckman Coulter). Incremental production of ATP was expressed as a percentage of control.

### Radionuclide uptake studies

The procedures of iodide uptake were previously described [4]. Cells (1 × 10^5^ cells/well) were seeded in 24-well plates and then treated accordingly. ^131^I solution (Atom Gaoke, Beijing, China) (175 KBq) was added and the cells were incubated for 1 h at 37 °C. Cells were washed twice with ice-cold phosphate-buffered saline (PBS) and dissolved in 0.1% NaOH. Cell lysates were collected and cellular radioactive accumulation was measured by a gamma counter (Zonkia Scientific Instruments Inc., Anhui, China). For determining the radionuclide uptake in relation to incubation time, cells were cultured with 175 KBq ^131^I for 5, l0, 20, 30, 60, 90 and 120 min. And radionuclide absorption was determined as above.

### Tumor cell xenograft model

Male nude mice (4–6 weeks of age) were purchased from Xiangya Medical College Animal Laboratory (Changsha, China). All experiments were approved by our institutional Animal Ethics Committee. The animals were raised in pathogen-free conditions with a 12-h light-dark cycle with an ad libitum supply of water and food. At one week after adaptive feeding, tumor model was established by a subcutaneous injection of 200 μL of sterile PBS containing 1 × 10^6^ cells FTC-133 or TPC-1 cell transfected with HMGB1 shRNA or control shRNA via right armpit. As previously described [30], food was withdrawn at 18 h, but not water, before experiment when murine tumors grew > 150 mm^3^. For each experiment, 20 mice were randomly divided into the following four groups: (a) control shRNA model without hunger (vehicle group); (b) control shRNA model with hunger (hunger group); (c) HMGB1 shRNA model without hunger (vehicle group) and (d) HMGB1 shRNA model with hunger (hunger group).

### In vivo tumor imaging

After establishing a xenograft tumor model, nude mice received a tail injection of 37 MBq of 99mTCO4 ^-^. Static SPECT images were acquired after 10 min using a gamma camera (GE Healthcare, Waukesha, WI, USA) with a low-energy high-resolution collimator.

### Biodistribution

Nude mice were sacrificed at the end of SPECT scan (90-min timepoint). Blood, heart, liver, spleen, lung, thyroid, stomach and tumor tissues were harvested and weighed. And radioactivity was measured by a gamma counter and the corresponding CPM count/mg tissue calculated.

### Statistical analysis

All statistical analyses were performed using SPSS 19.0 software (IBM Corp, Armonk, NY, USA). And the results were expressed as mean ± standard deviation (SD). Group means were compared by Student’s *t*-test for independent data. All *P-*values were two-tailed and *P* < 0.05 was deemed as statistically significant.

## Results

### HMGB1 expression is up-regulated in thyroid cancer and associated with clinicopathologic features

**Fig. 1:**
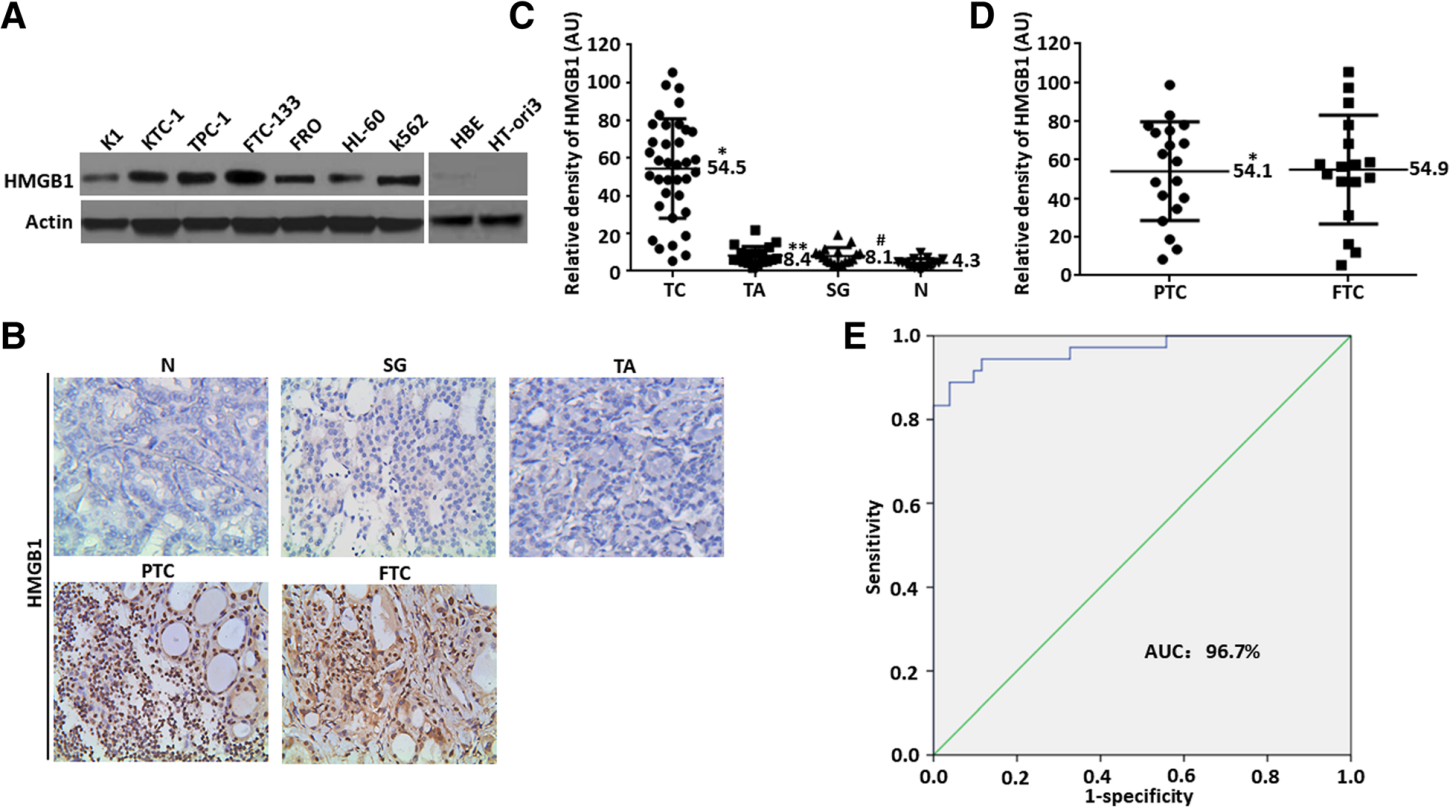
HMGB1 expression became up-regulated in thyroid cancer and was associated with clinicopathologic features (**a**) Western blot of HMGB1 and actin in various cell lines hinted at an over-expression of HMGB1 in thyroid cancer; (**b**) Immunohistochemical staining of HMGB1 was performed for different tissues. TC, thyroid cancer; TA, thyroid adenoma; SG, simple goiter; N, normal thyroid; PTC, papillary thyroid carcinoma; FTC, follicular thyroid carcinoma; (**c**) Relative expression levels of HMGB1 in different tissues. Total mRNA was extracted from normal or patient tissues and HMGB1 level determined by relative optical intensity (in arbitrary units, AU) of bands on RT-PCR. Each dot represented relative level of HMGB1 in each individual sample. *P < 0.01 vs. normal thyroid; **P > 0.01 vs. normal thyroid; #*P* > 0.01 vs. normal thyroid; (**d**) Relative expression levels of HMGB1 in thyroid cancer. Total mRNA was extracted from thyroid cancer patients’ tissues and HMGB1 level determined by relative optical intensity (in arbitrary units, AU) of bands on RT-PCR. Each dot represented relative level of HMGB1 in an individual sample. *P > 0.01 vs. FTC; (**e**) HMGB1 expression level for differentiating thyroid cancer tissues from non-thyroid cancer tissues in our validated cohort; AUC: 96.7%, sensitivity: 88.9% and specificity: 96.2% in the validated cohort

Firstly the levels of HMGB1 expression were determined by Western blot in different cell lines. Similar to leukemia (HL-60 & K562) and HBE cells in our previous studies [22, 24], HMGB1 expression was high in all five thyroid cancer cell lines (K1, KTC-1, TPC-1, FTC-133 & FRO) and yet low in normal human thyroid cells HT-ori3 (Fig. 1a). For further evaluating the clinical relevance, HMGB1 expression was detected by immunohistochemistry and RT-PCR in thyroid cancer samples from 36 patients with thyroid cancer [19 patients with papillary thyroid carcinoma (PTC) and 17 patients with follicular thyroid carcinoma (FTC)], 20 patients with thyroid adenoma, 17 patients with simple goiter and 15 patients with normal thyroid. Higher levels of HMGB1 expression were detected in thyroid cancer samples (Fig. 1b & c), but not in samples from thyroid adenoma, simple goiter or normal thyroid (Fig. 1b & c). Meanwhile, there was no significant difference as compared with the expression of PTC/FTC (Fig. 1d). Then a ROC curve was plotted for evaluating the diagnostic potential of HMGB1 expression level. In our validated cohort (36 cases of thyroid cancer tissues and 52 cases of non-thyroid cancer tissues), ROC curve analysis indicated that HMGB1 expression level might be a promising marker for differentiating malignant thyroid cancer from normal tissue with a sensitivity of 88.9% and a specificity of 96.2% (an area under the ROC curve: 96.7%; Fig. 1e). Thus HMGB1 may play an important role in tumorigenesis.

Furthermore, the relationship was examined between relative expression of HMGB1 with clinical features. Based on 36 PTC/FTC tissues, the patients were classified into low and high-expression groups according to the median value. The results indicated that tumor size, lymph node metastasis and clinical stage were significantly correlated with high HMGB1 expression (Table 1). However, no significant association existed between HMGB1 expression and gender, age or histological type. Thus HMGB1 reflected different clinicopathologic features in thyroid cancer.

### HMGB1 regulated autophagy in thyroid cancer cells

**Fig. 2:**
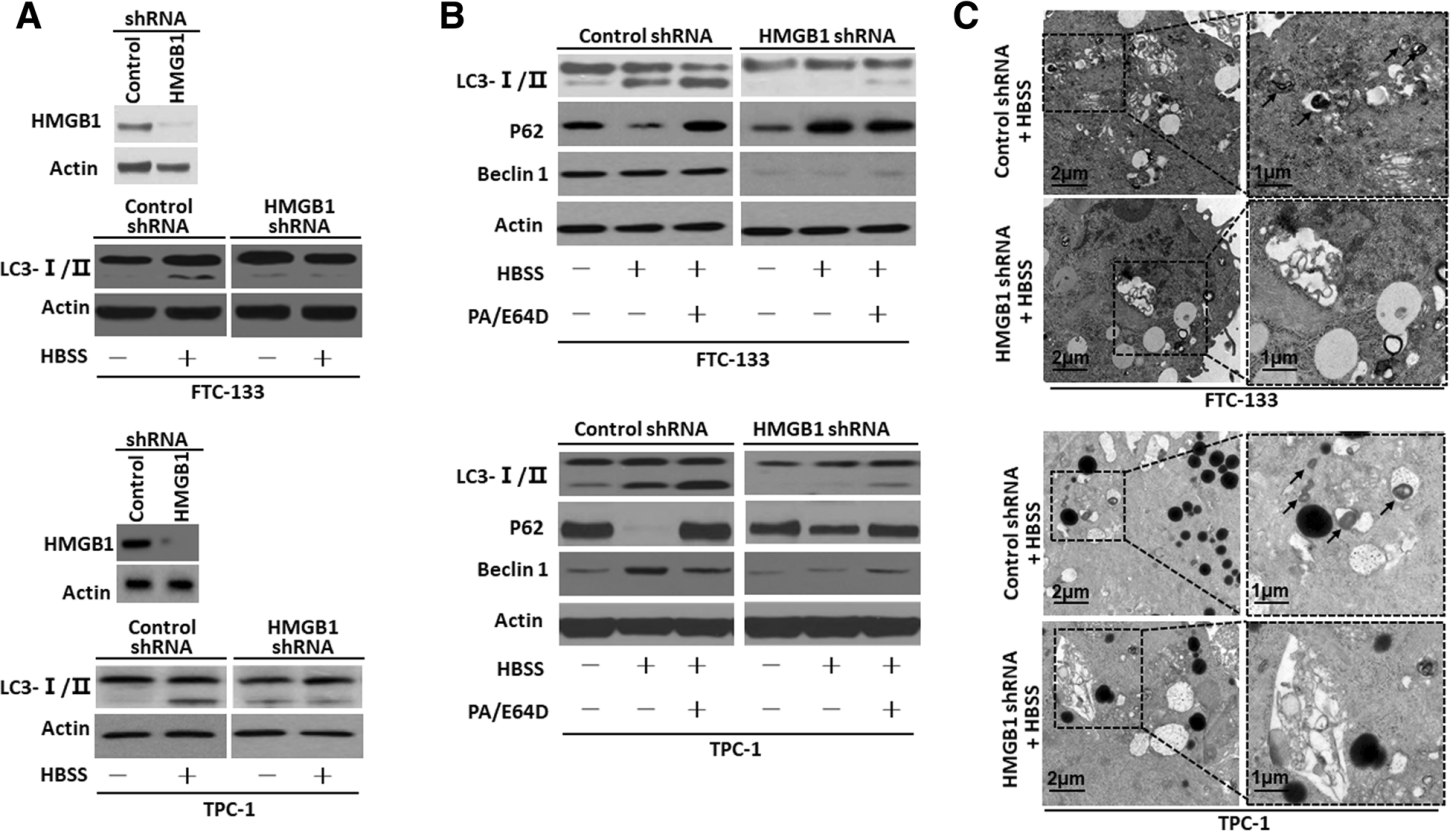
HMGB1 regulated autophagy in thyroid cancer cells (**a**) FTC-133/TPC-1 cells were transfected with HMGB1 shRNA and control shRNA and then starved by HBSS for 2 h. And LC3-I/II level was assayed by Western blot; (**b**) FTC-133/TPC-1 cells were transfected with HMGB1 shRNA and control shRNA and then pre-treated for 1 h with pepstatin A (PA, 10 μM) and E64D (10 μM) as indicated. Cells were subsequently treated for 3 h with HBSS in continuous presence or absence PA/E64D inhibitors. LC3-I/II, Beclin1 and p62 levels were assayed by Western blot; (**c**) Ultrastructural features in FTC-133/TPC-1 cells transfected with HMGB1 shRNA and control shRNA after a 3-h treatment of HBSS. More autophagosomes were seen in control shRNA plus HBSS-treated cells than in cells treated with HMGB1 shRNA plus HBSS. Arrows indicated autophagosomes

Mounting evidence has indicated that autophagy plays an important role in the regulation of cancer development and progression and in determining the response of tumor cells to anticancer therapy [31]. Firstly immunohistochemistry was employed for detecting Beclin1, a protein formed autophagosomes during autophagic sequestration. Similar to HMGB1 expression, the levels of Beclin1 expression were high in thyroid cancer tissues, but not in tissues derived from thyroid adenoma, simple goiter or normal thyroid (Additional file 2: Figure S2). For examining whether or not HMGB1 is a direct activator of autophagy, immunoblot was employed for measuring microtubule-associated protein light chain 3 (LC3) and its conversion products (LC3-I to LC3-II) [32]. With a depletion of HMGB1 expression, the classical autophagic stimuli HBSS down-regulated the expression of LC3-II versus control group (Fig. 2a).

For examining whether or not HMGB1 regulates autophagic flux, we evaluated the expression of SQSTM1/sequestosome 1 (p62), a long-lived scaffolding protein as a substrate of autophagy, is involved in transporting ubiquitinated protein destined for proteasomal digestion [33]. A knockdown of HMGB1 lowered the levels of LC3-II and Beclin1, yet boosted the level of p62 as compared with control shRNA group, implying that p62 degradation is dependent on HMGB1-induced autophagy (Fig. 2b). Moreover, a treatment of lysosomal protease inhibitors pepstatin and E64D up-regulated the expressions of LC3-II and p62 in control shRNA group, but not in HMGB1 shRNA group (Fig. 2b). These results indicated that an elevation of LC3-II was not due to reduced degradation of lipidated LC3. Rather it was a result of enhanced autophagic flux. Furthermore, there was a marked decrease of LC3 puncta formation after HMGB1 depletion by immunofluorescent analysis (Additional file 3: Figure S3). Finally ultrastructural analysis provided the most convincing evidence of autophagy. After a depletion of HMGB1, thyroid cancer cells exhibited fewer autophagosomes as compared with control shRNA group (Fig. 2c), supporting a critical role of HMGB1 in regulating autophagy.

### HMGB1-mediated autophagy regulates NIS expression and iodide uptake

**Fig. 3:**
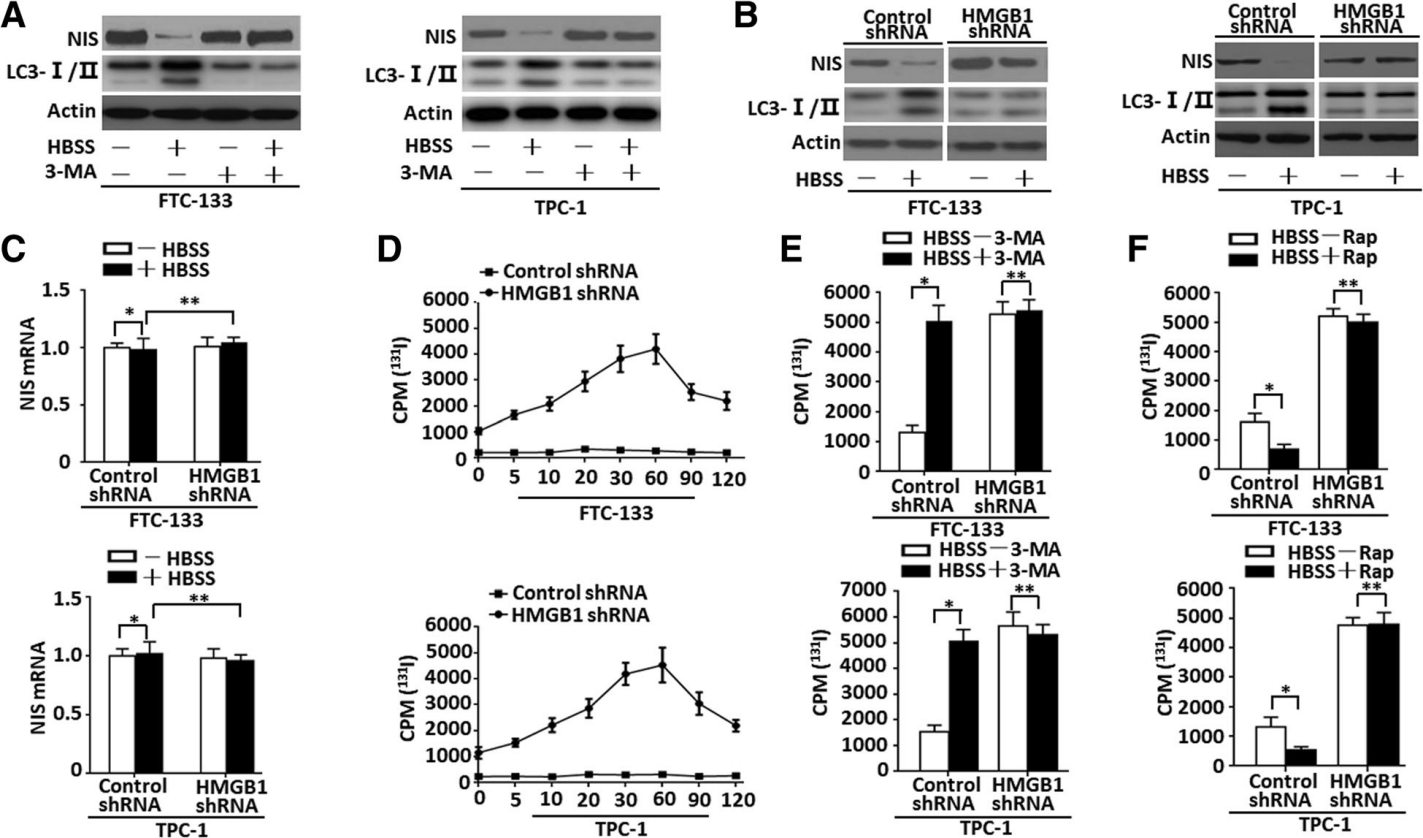
HMGB1-mediated autophagy regulated NIS expression and iodide uptake (**a**). FTC-133/TPC-1 cells were pretreated with 3-MA (10 mM) treatment for 1 h and then starved by HBSS for 3 h. LC3-I/II and NIS levels were assayed by Western blot; (**b**&**c**) FTC-133/TPC-1 cells were transfected with HMGB1 shRNA and control shRNA and then starved by HBSS for 3 h. LC3-I/II and NIS levels were assayed by Western blot. And NIS mRNA was assayed by qRT-PCR (*n* = 3, *P > 0.01, **P > 0.01); (**d**) Dynamic uptaking: FTC-133/TPC-1 cells were transfected with HMGB1 shRNA and control shRNA and starved by HBSS for 3 h. Indicated cells were cultured with 175 KBq ^131^I for 5, l0, 20, 30, 60, 90 and 120 min. The uptake of ^131^I in indicated cells was detected by a gamma counter; (**e**) Radionuclide uptaking: FTC-133/TPC-1 cells were transfected with HMGB1 shRNA and control shRNA in the presence or absence of 3-MA (10 mM) treatment for 1 h and then starved by HBSS for 3 h. After 1-h incubating with ^131^I, the uptake of ^131^I in indicated cells was detected by a gamma counter (*n* = 3, *P < 0.01, **P > 0.01); (**f**) FTC-133/TPC-1 cells transfected with HMGB1 shRNA and control shRNA were starved by HBSS for 3 h and then treated with rapamycin (1 μM) for 12 h. After 1-h incubating with ^131^I, the uptake of ^131^I in indicated cells was detected by a gamma counter. Rap, rapamycin. (*n* = 3, *P < 0.01, **P > 0.01)

NIS-mediated iodide uptake plays an important physiological role in the thyroid gland function, and is important for the diagnosis and treatment of Graves’ disease and thyroid cancer [2]. Study demonstrated that NIS protein is degraded in mammary cells when the autophagy-lysosome pathway is activated [18]. To investigate the effect of autophagy on NIS expression, we first analyzed the relation between LC3 conversion (LC3-I to LC3-II) with NIS expression by western blot. A treatment of HBSS boosted LC3-II level, but significantly lowered the NIS level as compared with untreated group (Fig. 3a). Furthermore, we inhibited autophagy by using 3-MA, a PI3K inhibitor, which blocks the formation of autophagosomes and inhibits autophagy dependent protein degradation. A treatment of 3-MA significantly lowered the expression of LC3-II and NIS protein degradation (Fig. 3a). Moreover, autophagy was suppressed by Spautin-1, an inhibitor of autophagy blocking the peptidase activity of ubiquitin carboxyl-terminal hydrolase 10 (USP10) and ubiquitin carboxyl-terminal hydrolase 13 (USP13), leading to heightened ubiquitination and proteasomal degradation of Beclin-1-containing class III PI3K complexes [34]. A treatment of Spautin-1 also significantly reduced the expression of LC3-II and NIS protein degradation (Additional file 4: Figure S4). For examining whether or not HMGB1-mediated autophagy regulated NIS degradation, the expressions of LC3-II and NIS were detected in HMGB1-knockdown cells. A cellular knockdown of HMGB1 expression suppressed HBSS-induced autophagy and NIS protein degradation as compared with control shRNA group (Fig. 3b), but not for a down-regulation of NIS mRNA expression (Fig. 3c). Thus HMGB1-mediated autophagy is a critical regulator for NIS degradation rather than an up-regulation of NIS mRNA.

Thyroid imaging using ^131^I has played a key role in diagnosing and treating thyroid cancer [3]. As a transmembrane glycoprotein at basolateral pole in thyroid follicular cells, NIS mediates active iodide uptake in thyroid gland [2]. In HBSS-induced HMGB1 knockdown cells, uptaking of ^131^I rapidly reached a plateau at 30–60 min, but not in control cells (Fig. 3d). To further determining the impact of autophagy on iodide uptake, cells were incubated with HBSS and autophagy inhibitors (e.g. 3-MA or Spautin-1) or an autophagy inducer (e.g. rapamycin) and then gamma counter analysis was performed. Co-treatment of 3-MA/Spautin-1 and HBSS boosted the uptake of ^131^I in control group cells while it had no effect upon the uptake of ^131^I in HMGB1 knockdown cells compared with HBSS treated alone (Fig. 3e and Additional file 4: Figure S4). Meanwhile, a co-treatment of HBSS and rapamycin had no effect upon the uptake of ^131^I in HMGB1 knockdown cells as compared with HBSS treatment alone (Fig. 3f). Thus HMGB1 was required for ^131^I uptake in HBSS-treated thyroid cancer cells.

### ROS was sufficient for inducing HMGB1 translocation and enhancing autophagy

**Fig. 4:**
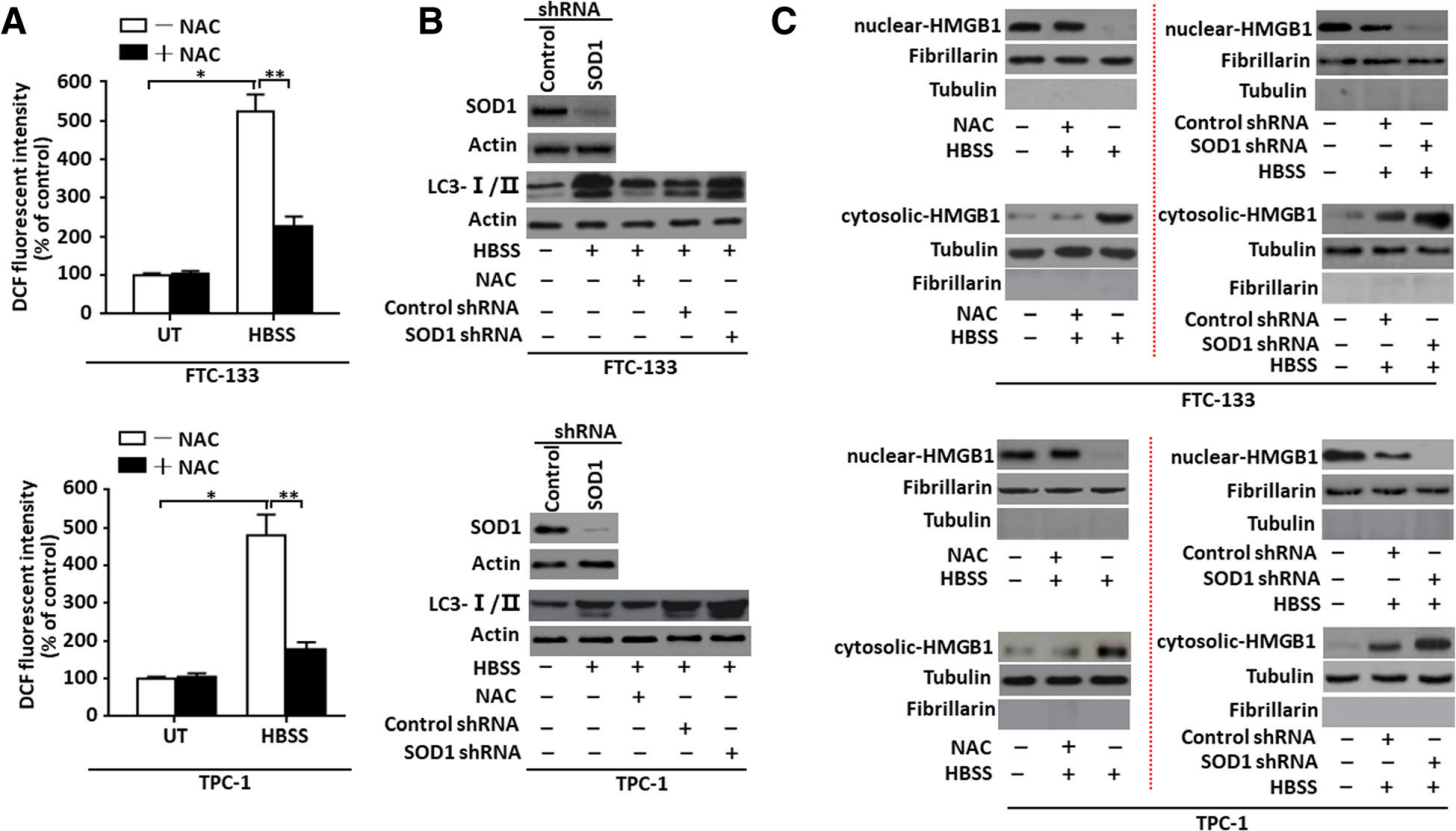
ROS was sufficient for inducing HMGB1 translocation and enhancing autophagy (**a**) FTC-133/TPC-1 cells were pretreated with the antioxidant (NAC, 2 mM) for 1 h and then starved by HBSS for 3 h. ROS production was assessed by measuring the fluorescent intensity of DCF on a fluorescent plate reader. Incremental production of ROS was expressed as a percentage of control (*n* = 3, *P < 0.01, **P < 0.01). UT, untreated group; (**b**) Antioxidant and SOD1 RNAi regulated starvation-induced autophagy as measured by LC3-II expression. FTC-133/TPC-1 cells were pretreated with NAC (2 mM) for 1 h or SOD1 RNAi for 48 h, and then starved by HBSS for 3 h. LC3-I/II level was assayed by Western blot. (**c**) Antioxidant and SOD1 RNAi regulated starvation-induced HMGB1 translocation. FTC-133/TPC-1 cells were pretreated with NAC (2 mM) for 1 h or SOD1 RNAi for 48 h, and then starved by HBSS for 3 h. And the expression of nuclear/cytosolic HMGB1 was assayed by Western blot. Fibrillarin was a nuclear fraction control and tubulin a cytoplasmic fraction control

ROS function as signaling molecules of regulating both cell survival and death through various pathways. Many stimuli that induce ROS generation, such as nutrient starvation, mitochondrial toxins, and hypoxia, also induce autophagy in cell survival, death, development and many human diseases [35]. Here, we found that a treatment of HBSS induced ROS generation, whereas a pretreatment of a ROS quencher NAC significantly decreased ROS levels, suggesting that HBSS was a valid stimulus for ROS generation in thyroid cancer cells (Fig. 4a). For evaluating the relationship between ROS and autophagy in thyroid cancer cells, LC3 conversion (LC3-I to LC3-II) was detected at different ROS levels. A knockdown of superoxide dismutase 1 (SOD1), functioning as a ROS scavenger, enhanced HBSS-induced LC3 conversion (Fig. 4b). In contrast, NAC suppressed HBSS-induced LC3-II expression (Fig. 4b), implying that ROS regulated the autophagy of thyroid cancer cells.

HMGB1 protein is both a nuclear DNA binding factor and a secreted protein and its activities are determined by its intracellular localization and posttranslational modifications [20]. Classical autophagic stimuli, such as starvation (HBSS) or rapamycin, promoted HMGB1 translocation from nucleus to cytosol and resulted in enhanced autophagy after sustained cellular stress [21]. To evaluating whether HMGB1 translocation was regulated by ROS during HBSS-induced autophagy, thyroid cancer cells were pretreated with either NAC or SOD1 RNAi. It was found that HMGB1 was predominantly located in nucleus under normal conditions, whereas it was noticeably high in cytosol under HBSS stimuli (Fig. 4c). A pretreatment of NAC in thyroid cancer cells blocked HMGB1 translocation from nucleus into cytosol (Fig. 4c). In contrast, a knockdown of SOD1 expression boosted HMGB1 translocation (Fig. 4c). Thus ROS was sufficient for inducing HMGB1 translocation and sustaining autophagy in thyroid cancer cells.

### ROS/AMPK/mTOR pathway was required for HMGB1-mediated autophagy regulating NIS expression

**Fig. 5:**
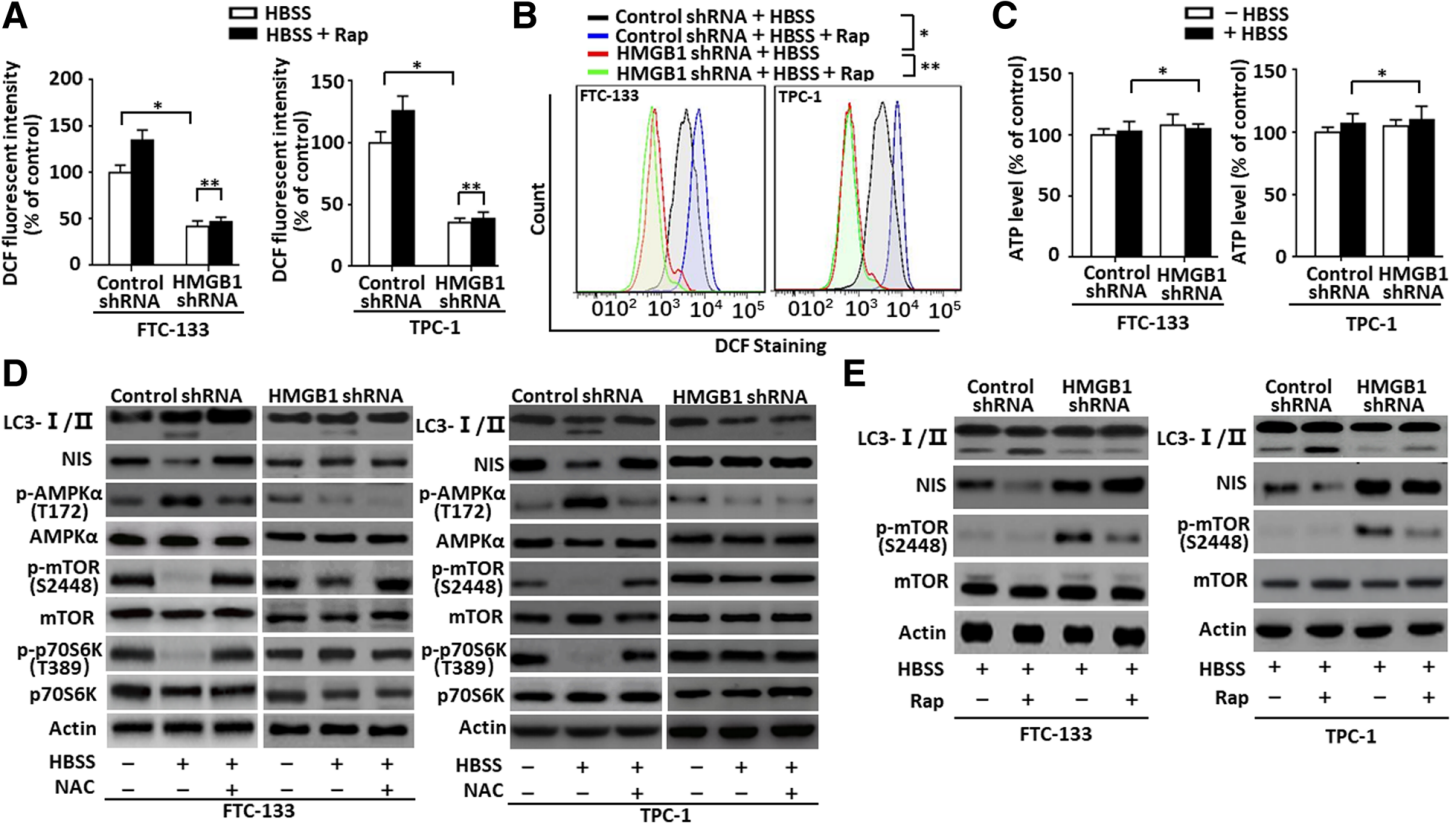
ROS/AMPK/mTOR pathway was required for HMGB1-mediated autophagy regulating NIS expression (**a**) FTC-133/TPC-1 cells transfected with HMGB1 shRNA and control shRNA transfection were starved by HBSS for 3 h and then treated with rapamycin (1 μM) for 12 h. ROS production was assessed by measuring the fluorescent intensity of DCF on a fluorescent plate reader. Incremental production of ROS was expressed as a percentage of control. Rap, rapamycin. (*n* = 3, *P < 0.01, **P > 0.01); (**b**) FTC-133/TPC-1 cells transfected with HMGB1 shRNA and control shRNA were starved by HBSS for 3 h and then treated with rapamycin (1 μM) for 12 h. Flow cytometry was performed for measuring the ROS level by a DCFH-DA probe in indicated cells. Rap, rapamycin. (*n* = 3, *P < 0.01, **P > 0.01); (**c**) FTC-133/TPC-1 cells were transfected with HMGB1 shRNA and control shRNA and then starved by HBSS for 3 h. ATP levels were detected by ATP Assay Kit (*n* = 3, *P > 0.01); (**d**) FTC-133/TPC-1 cells were pretreated with NAC (2 mM) and then starved by HBSS for 3 h. LC3-I/II, NIS, p-AMPK, AMPK, p-mTOR, mTOR, p-p70S6K and p70S6K levels were assayed by Western blot; (**e**) FTC-133/TPC-1 cells transfected with HMGB1 shRNA and control shRNA were starved by HBSS for 3 h and then treated with rapamycin (1 μM) for 12 h. LC3-I/II, NIS, p-mTOR and mTOR levels were assayed by Western blot. Rap, rapamycin

Increasing evidence points to ROS as important activators of autophagy [21, 22]. Nutrient deprivation is a canonical activator of autophagy, and under starvation conditions, ROS are produced and accumulate [21]. ROS was higher in HBSS-treated cells and sufficient for inducing HMGB1 translocation and sustaining autophagy (Fig. 4). For examining whether HMGB1-mediated autophagy regulated ROS generation, the ROS levels were measured in HBSS-induced cells with or without rapamycin treatment. A knockdown of HMGB1 expression significantly suppressed HBSS-induced ROS generation as compared with control shRNA group and rapamycin failed to reverse inhibition in HMGB1 knockdown cells (Fig. 5a). Then ROS levels were quantified through flow cytometry after staining with a DCFH-DA probe. It indicated that ROS levels were markedly lowered after a treatment of HBSS in HMGB1-knockdown FTC-133/TPC-1 cells (Fig. 5b). Additionally, a co-treatment of HBSS and rapamycin also failed to reverse an inhibition of ROS generation in HMGB1-knockdown cells (Fig. 5b), implying that HMGB1 is an important regulator for HBSS-induced ROS generation.

Functioning as a metabolic checkpoint, AMPK is typically activated by a higher AMP/ATP ratio for maintaining energy homeostasis [36]. It was previously assumed that AMPK was activated by a higher AMP/ATP ratio due to cellular and environmental stressors. However, recent studies have reported that ROS also exerted an evident impact on the activation of AMPK under certain conditions [37]. And ROS levels declined markedly in HMGB1-knockdown cells (Fig. 5a). Our data further revealed that ATP level became almost constant between control and HMGB1 shRNA groups (Fig. 5c), implying that ATP was not involved in HMGB1-mediated HBSS-induced autophagy in FTC-133/TPC-1 cells.

For further examining whether AMPK and ROS are vital for HMGB1-mediated autophagy, AMPK phosphorylation (p-AMPK), LC3 level and NIS degeneration were detected in HBSS-induced cells with or without ROS scavenger (NAC). HBSS treatment significantly boosted LC3-II and p-AMPK levels and down-regulated NIS expression in control shRNA group while NAC suppressed HBSS-induced LC3 conversion, phosphorylation of AMPK and NIS degeneration (Fig. 5d). Moreover, a depletion of HMGB1 expression arrested LC3 conversion, phosphorylation of AMPK and NIS degeneration as compared with control shRNA group and NAC had no contribution to these proteins impression in HMGB1-knockdown cells (Fig. 5d). As demonstrated by many studies, activated AMPK could inactivate mTOR [38], a kind of conserved serine/threonine protein kinases, and thus promoting the induction of autophagy. Contrary to p-AMPK expression, HBSS treatment suppressed the phosphorylation of mTOR (p-mTOR) and p70S6K (p-p70S6K) in control shRNA group cells while NAC reserved the inhibited phosphorylations of mTOR and p70S6K (Fig. 5d). Meanwhile, a depletion of HMGB1 expression boosted the levels of p-mTOR and p-p70S6K as compared with control shRNA group. And NAC also had no contribution to these proteins impression in HMGB1-knockdown cells (Fig. 5d). Furthermore, mTOR inhibitor (classical autophagic stimuli), rapamycin, accelerated HBSS-induced LC3 conversion and NIS degeneration in control group, but not in HMGB1-knockdown cells (Fig. 5e). Thus HMGB1-mediated autophagy regulated NIS expression through a ROS/AMPK/mTOR pathway.

### HMGB1-mediated autophagy regulated the imaging of ^99m^TcO4^−^ and AMPK/mTOR pathway in tumor-bearing nude mice in vivo

**Fig. 6:**
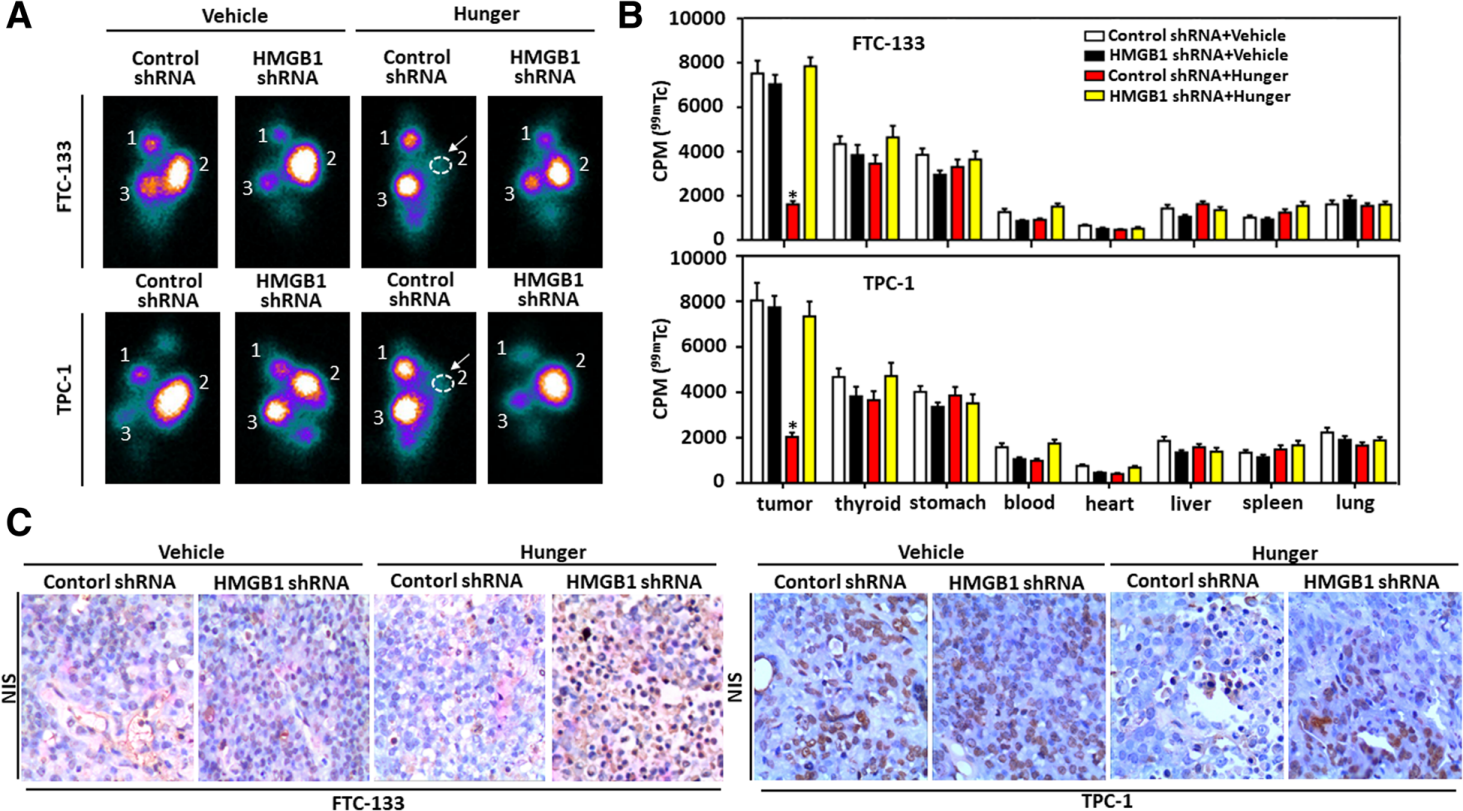
HMGB1-mediated autophagy regulates the imaging of ^99m^TcO4^−^ and NIS expression in tumor-bearing nude mice in vivo (**a**) Nude mice received a subcutaneous injection of HMGB1 shRNA and control shRNA cells and then food was withdrawn at 18 h before experimentation when murine tumor was > 150 mm^3^. Wholebody scintigraph of bearing-tumor mice was performed at 10 min after a tail injection of ^99m^Tc-pertechnetate. 1: thyroid gland, 2: transplanted tumor, 3: stomach. Arrows indicated that transplanted tumor was not visualized; (**b**) Biodistribution of ^99m^TcO4 ^-^ in tumor-bearing nude mice (*n* = 3, *P < 0.01 vs. HMGB1 shRNA hunger group or control shRNA vehicle group); (**c**) Immunohistochemical staining of NIS was performed with isolated tumor at the end of experiment

For examining whether or not a suppression of HMGB1 expression affected NIS expression and iodide uptake in vivo, indicated cells (control shRNA or HMGB1 shRNA-transfected cells) were subcutaneously introduced into right flank of nude mice (weight, approximately 20 g). Food was withdrawn at 18 h before experiment (hunger group) when nude mice grew tumors > 150 mm^3^. After establishing this xenograft tumor model, SPECT was performed on all nude mice after an intravenous injection of ^99m^TcO4^−^. The 10-min static SPECT showed that tumor tissue of vehicle group accumulated ^99m^TcO4^−^ significantly, leading to scintigraphic visualization (Fig. 6a). The accumulation of ^99m^TcO4^−^ decreased in control shRNA cells formed tumors of hunger group and a knockdown of HMGB1 reversed an accumulation of ^99m^TcO4^−^ (Fig. 6a). Normal NIS-expressing tissues, including thyroid gland and stomach, were also distinctly visible. After SPECT scaning, blood, heart, liver, spleen, lung, thyroid, stomach and tumor tissues were harvested and weighed and biodistribution was detected by a gamma counter. The data showed that the uptake of ^99m^TcO4^−^ decreased significantly in control shRNA cells formed tumors of hunger group, but not in tumors of other groups (Fig. 6b). Except for thyroid gland and stomach, the uptake of ^99m^TcO4- was significantly low in other organs (Fig. 6b). Furthermore, both immunohistochemistry and Western blot revealed that NIS was weakly expressed in hunger group tumors formed by control shRNA-transfected cells, but not in other groups (Fig. 6c & Additional file 5: Figure S5). Similar to NIS expression, p-mTOR expression also decreased in hunger group tumors formed by control shRNA-transfected cells as compared with others, whereas p-AMPK and Beclin1 levels rose (Additional file 5: Figure S5). Thus HMGB1-mediated autophagy promoted NIS degradation and lowered iodide uptake in vivo*.*

## Discussion

Radioiodide accumulation in thyroid tissue has been exploited clinically for diagnosing, treating and following up thyroid pathologies for several decades. The capability of thyroid epithelia of concentrating I^−^ is ultimately dependent on functional NIS expression at plasma membrane. NIS has been used as a therapeutic gene approach for treating cancers through its ability of concentrating therapeutic doses of radionuclide in target cells [2, 3]. Our study implied that HMGB1 was directly involved in the positive regulation and maintenance of autophagy in HBSS-treated thyroid cancer cells, thereby promoting NIS degradation and lowering iodide uptake. A depletion of HMGB1 suppressed starvation-induced NIS degradation and boosted iodide uptake in vitro and in vivo through a ROS/AMPK/mTOR-dependent pathway. Thus our findings may provide novel therapeutic options for thyroid cancer patients already on radioiodide therapy.

HMGB1, an abundant nonhistone protein, has been shown to play a central role in inducing autophagy. And HMGB1-induced autophagy was essential for drug resistance of osteosarcoma, leukemia, lung cancer and breast cancer [39]. Recent studies have implied that autophagy was involved in several steps of thyroid tumor initiation and progression as well as in therapeutic resistance and therefore it could be exploited for therapeutic applications [40]. However, HMGB1 expression and its function in mediating autophagy in thyroid cancer have been poorly elucidated. Here HMGB1 expression became up-regulated in samples derived from patients of PTC, FTC and thyroid cancer cell lines. Conversely, their levels were lower in thyroid adenoma, simple goiter and normal thyroid samples. Furthermore, its expression levels were correlated with clinicopathologic features of thyroid cancer patients. Higher related to lymph node metastasis risk, later clinical stage and tumor size, suggesting a potential contributory role of HMGB1 in tumorigenesis of thyroid cancer.

Recently mounting evidence has implied that NIS post-transcriptional regulation is an important mechanism during an uptake of iodide. Riedel et al. [10] have showed that the presence of NIS post-translational regulation through its cellular traffic and stability, thus consequently regulating iodide uptake by thyrocytes. As an important post-transcriptional mechanism, autophagy-mediated processes regulated NIS protein degradation. NIS protein became degraded in mammary cells after an activation of autophagy-lysosome pathway through an inhibition of MEK [18]. As reported by Cazarin et al. [11], AMPK activation might regulate NIS protein degradation through an autophagy-lysosome pathway in rat thyroid cells. It hinted at a potential contributory effect of autophagy on NIS protein degradation. Our previous studies have confirmed that HMGB1 is a positive regulator of autophagy in leukemic cells, rendering leukemic cells resistant to chemotherapeutic drugs. Here HBSS treatment induced autophagy of thyroid cancer cells and a targeted deletion of HMGB1 cancelled out this starvation-induced autophagy. Additionally, HBSS-induced autophagy lowered NIS expression and iodide uptake and using autophagy inhibitors blocked the HMGB1-mediated regulation of starvation-induced autophagy and boosted iodide uptake. In contrast, rapamycin, an autophagy inducer, enhanced the HMGB1-mediated regulation of starvation-induced autophagy and reduced iodide uptake. However, NIS expression and iodide uptake showed no reduction in HMGB1 knockdown cells after a co-treatment of 3-MA or rapamycin. It hinted at a dominant role of HMGB1 in regulating autophagy and NIS expression and iodide uptake were highly correlated with autophagy.

Both ROS and autophagy play important roles in cellular stress responses through a variety of complicated signaling pathways and molecules. Many stimuli of inducing ROS generation, such as nutrient starvation, mitochondrial toxins and hypoxia, also induce autophagy [41]. Tang et al. [21] have showed that ROS generated during starvation and rapamycin treatment served as signaling molecules of initiating autophagy and promoting HMGB1 cytosolic translocation from nucleus. Our experimental data also indicated that HMGB1 translocation during autophagy was ROS-dependent in thyroid cancer cells. Pharmacological inhibition of ROS production suppressed HBSS-induced autophagy, whereas a knockdown of SOD1 expression promoted HBSS-induced autophagy. Furthermore, a pre-treatment of NAC led to a decrease of HMGB1 cytosolic translocation while there was an increase with a depletion of SOD1 expression. These results suggested that ROS was sufficient for inducing HMGB1 translocation and sustaining autophagy in thyroid cancer cells. Compared with normal cells, both ROS and autophagy became altered in cancer cells. On one hand, ROS could induce autophagy through several distinct mechanisms involving a catalase activation of Atg4 and a disturbance of mETC [42]. On the other hand, defective autophagy increased oxidative stress in tumor cells [43]. Kang et al. [44] have showed HMGB1 was a crucial regulator of autophagy as a cellular defense mechanism in response to oxidative stress/injury. Here it was further confirmed that a knockdown of HMGB1 causing defective autophagy suppressed HBSS-induced ROS generation and a co-treatment of rapamycin failed to restore the ROS levels.

As an evolutionarily conserved serine/threonine kinase, AMPK plays a vital role in sustaining cellular metabolic balance and regulating NIS protein degradation [11, 45]. Both AMP/ATP ratio and ROS were essential for activating AMPK [37]. Here a knockdown of HMGB1 had no effect upon ATP levels, supporting that ROS, rather than ATP, was not involved in HMGB1-mediated autophagy induced by HBSS. Previous studies have examined the role of AMPK in thyroid disorder, such as nodular goiter and thyroid tumorigenesis. An activation of AMPK might be beneficial for thyroid diseases [46, 47]. And mTOR signaling pathway has been shown to play an important role in autophagy. Activating this pathway could suppress autophagy and enhance cellular growth and proliferation in both normal and tumor cells [38]. Studies have shown that activation of AMPK but inhibition of mTOR inducing autophagy has been regarded as an important mechanism for thyroid cancer therapy [48, 49]. Our previous study has demonstrated that HMGB1 is an intrinsic regulator of autophagy in leukemic cells through mTOR pathway [24]. In this study, we provide evidence that knockdown of HMGB1 inhibited starvation-induced autophagy, NIS degradation and AMPK phosphorylation in vitro and in vivo, but not for mTOR and P70S6K phosphorylation as well as NAC co-treatment in control group. Additionally, knockdown of HMGB1 restored the accumulated ^99m^TcO4^−^ of thyroid cancer cells xenograft after hunger treatment in nude mice. These preclinical results suggest that HMGB1-mediated autophagy regulating NIS degradation could be a potential target for radioiodide therapy in thyroid cancer.

## Conclusions

HMGB1 was over-expressed in thyroid cancer patients’ samples and cell lines and acted as a positive regulator of autophagy. ROS generated by cellular stress promoted HMGB1 cytosol translocation and sustained autophagy. HMGB1-mediated autophagy in starvation-treated cells regulated ROS generation and AMPK and mTOR phosphorylation, thereby regulating NIS protein degradation and iodide uptake. The discovery of HMGB1 as a critical regulator of autophagy has provided new insights into the molecular mechanism of NIS protein degradation for radioiodide therapy.

## Additional files

Additional file 1: **Figure S1** Verification of hNIS gene transfection in FTC-133 and TPC-1 cells (a) FTC-133 and TPC-1 cells were incubated with Ad-eGFP-hNIS for 48 h at a MOI of 800 and visualized under a fluorescent microscope (× 200). Untransfected cells had no expression of reporter protein. UT: untransfected group; (b) After transfecting with Ad-eGFP-hNIS for 48 h, NIS level was detected by RT-PCR and Western blot. Actin was a loading control. UT: untransfected group. (TIF 289 kb)

Additional file 2: **Figure S2** Beclin1 expression became up-regulated in thyroid cancer Immunohistochemical staining of Beclin1 was performed for different tissues. TA, thyroid adenoma; SG, simple goiter; N, normal thyroid; PTC, papillary thyroid carcinoma; FTC, follicular thyroid carcinoma. (TIF 489 kb)

Additional file 3: **Figure S3** Depletion of HMGB1 decreased LC3 puncta formation FTC-133/TPC-1 cells were transfected with HMGB1 shRNA and control shRNA and starved by HBSS for 3 h. LC3 puncta formation was detected by immunofluorescence under a confocal microscope. (TIF 409 kb)

Additional file 4: **Figure S4** Spautin-1 regulated NIS protein degradation and iodide uptake (a) FTC-133/TPC-1 cells were pretreated for 24 h with Spautin-1(10 μM) and then starved by HBSS for 3 h. LC3-I/II and NIS levels were assayed by Western blot; (b) FTC-133/TPC-1 cells were transfected with HMGB1 shRNA and control shRNA in the presence or absence of Spautin-1 (10 μM) treatment for 24 h and then starved by HBSS for 3 h. After 1-h incubation of ^131^I, the uptake of ^131^I in indicated cells was detected by a gamma counter (*n* = 3, *P < 0.01, **P > 0.01). (TIF 111 kb)

Additional file 5: **Figure S5** HMGB1-mediated autophagy regulated AMPK/mTOR pathway in tumor-bearing nude mice in vivo NIS, Beclin1, p-AMPK, AMPK, p-mTOR and mTOR levels were assayed by Western blot at the end of experiment. (TIF 138 kb)

## Data Availability

The datasets generated and analyzed during the current study are available from the corresponding author on reasonable request.

## Abbreviations

3-MA: 3-methyladenine
ALP: Autophagy-lysosome pathway
AMPK: AMP-activated protein kinase
DMEM: Dulbecco’s modified Eagle’s medium
DPI: Diphenyleneiodonium chloride
FBS: Fetal bovine serum
HBSS: Hank’s balanced salt solution
HMGB1: High-mobility group box 1
mTOR: mammalian target of rapamycin
NAC: N-acetylcysteine
NIS: sodium/iodide symporter
Rap: Rapamycin
ROS: Reactive oxygen species
SOD1: Superoxide dismutase 1
UPS: Ubiquitin-proteasome system
