# Supplementary figures and images for: HMGB1-mediated autophagy regulates sodium/iodide symporter protein degradation in thyroid cancer cells
Source: J Exp Clin Cancer Res. 2019 Jul 22;38:325. doi: 10.1186/s13046-019-1328-3 (PMC6647330; doi:10.1186/s13046-019-1328-3)

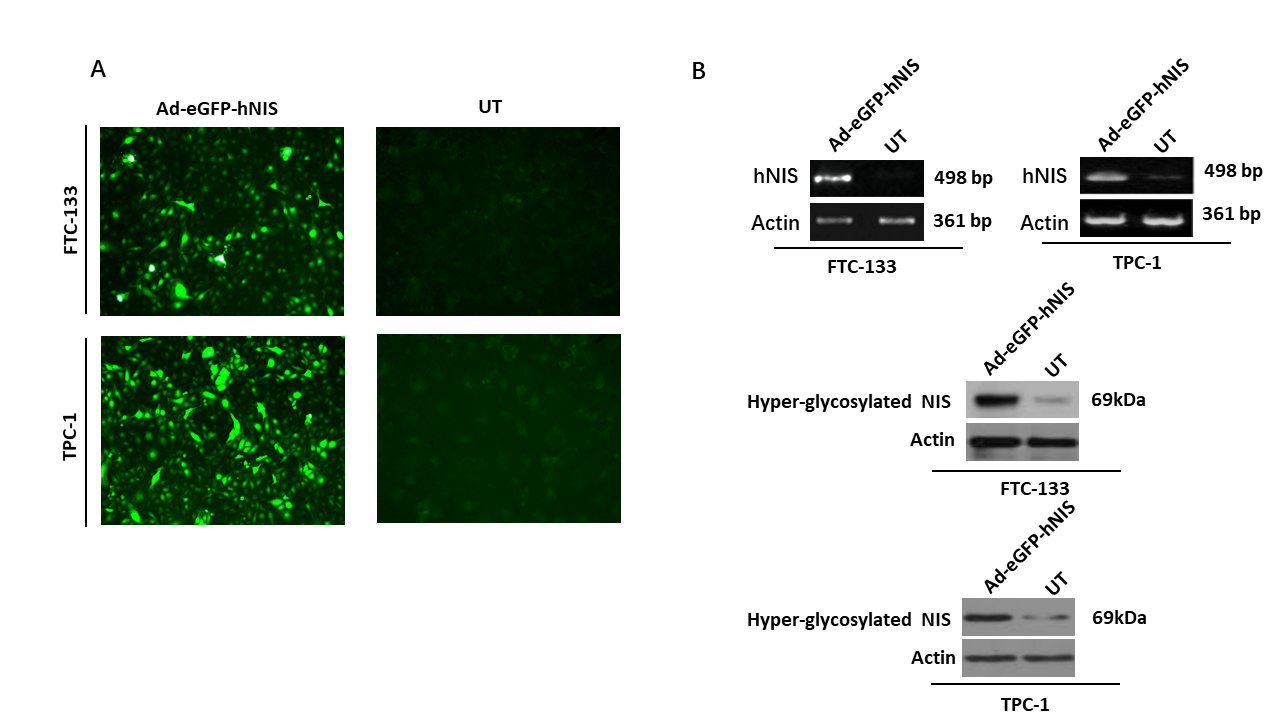

Supplement: Supplementary file 1 — Figure S1 Verification of hNIS gene transfection in FTC-133 and TPC-1 cells (a) FTC-133 and TPC-1 cells were incubated with Ad-eGFP-hNIS for 48 h at a MOI of 800 and visualized under a fluorescent microscope (× 200). Untransfected cells had no expression of reporter protein. UT: untransfected group; (b) After transfecting with Ad-eGFP-hNIS for 48 h, NIS level was detected by RT-PCR and Western blot. Actin was a loading control. UT: untransfected group. (TIF 289 kb) [file 13046_2019_1328_MOESM1_ESM.tif]

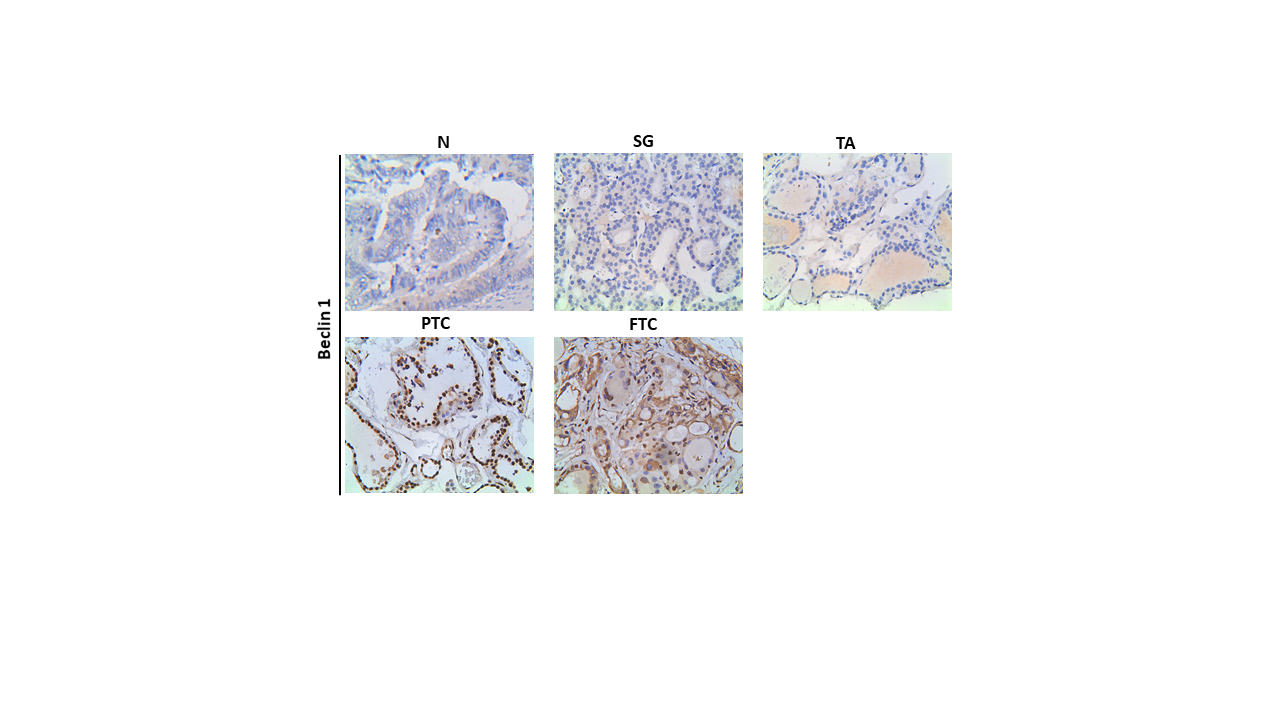

Supplement: Supplementary file 2 — Figure S2 Beclin1 expression became up-regulated in thyroid cancer Immunohistochemical staining of Beclin1 was performed for different tissues. TA, thyroid adenoma; SG, simple goiter; N, normal thyroid; PTC, papillary thyroid carcinoma; FTC, follicular thyroid carcinoma. (TIF 489 kb) [file 13046_2019_1328_MOESM2_ESM.tif]

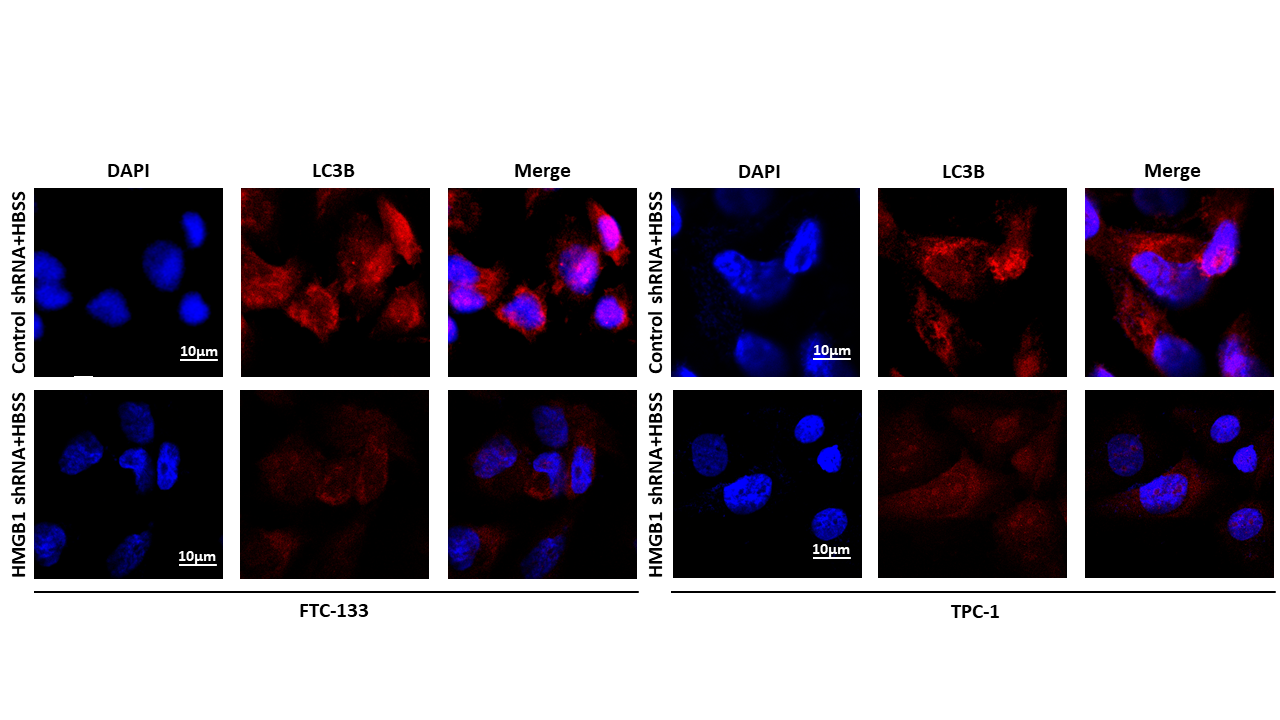

Supplement: Supplementary file 3 — Figure S3 Depletion of HMGB1 decreased LC3 puncta formation FTC-133/TPC-1 cells were transfected with HMGB1 shRNA and control shRNA and starved by HBSS for 3 h. LC3 puncta formation was detected by immunofluorescence under a confocal microscope. (TIF 409 kb) [file 13046_2019_1328_MOESM3_ESM.tif]

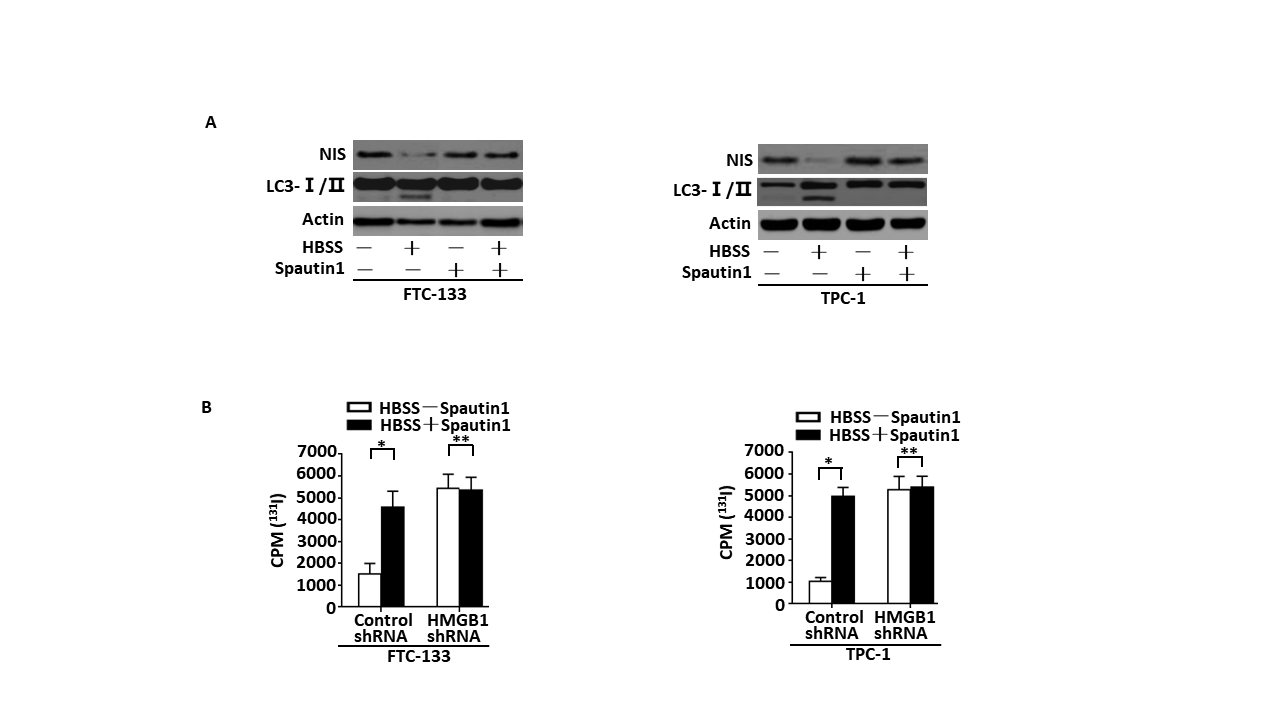

Supplement: Supplementary file 4 — Figure S4 Spautin-1 regulated NIS protein degradation and iodide uptake (a) FTC-133/TPC-1 cells were pretreated for 24 h with Spautin-1(10 μM) and then starved by HBSS for 3 h. LC3-I/II and NIS levels were assayed by Western blot; (b) FTC-133/TPC-1 cells were transfected with HMGB1 shRNA and control shRNA in the presence or absence of Spautin-1 (10 μM) treatment for 24 h and then starved by HBSS for 3 h. After 1-h incubation of 131I, the uptake of 131I in indicated cells was detected by a gamma counter (n = 3, *P < 0.01, **P > 0.01). (TIF 111 kb) [file 13046_2019_1328_MOESM4_ESM.tif]

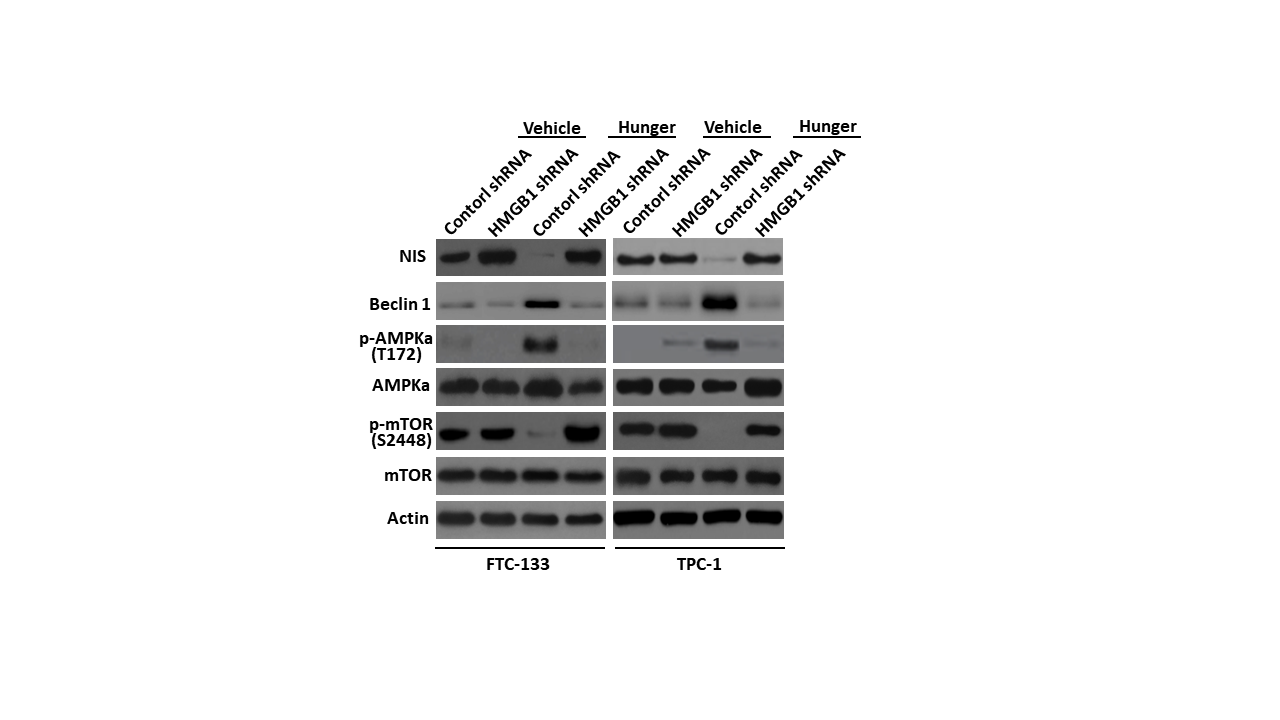

Supplement: Supplementary file 5 — Figure S5 HMGB1-mediated autophagy regulated AMPK/mTOR pathway in tumor-bearing nude mice in vivo NIS, Beclin1, p-AMPK, AMPK, p-mTOR and mTOR levels were assayed by Western blot at the end of experiment. (TIF 138 kb) [file 13046_2019_1328_MOESM5_ESM.tif]
